# A Case of Ovarian Hyperthecosis in a Postmenopausal Woman

**DOI:** 10.1210/jcemcr/luad148

**Published:** 2023-12-07

**Authors:** Saira Yousaf, Ryizan Nizar, Lawrence John, Aaron Simpson

**Affiliations:** Department of Diabetes and Endocrinology, Lister Hospital, East and North Hertfordshire NHS Foundation Trust, Stevenage SG1 4AB, United Kingdom; Department of Diabetes and Endocrinology, Great Western Hospitals NHS Foundation Trust, Swindon SN3 6BB, United Kingdom; Department of Pathology, Great Western Hospitals NHS Foundation Trust, Swindon SN3 6BB, United Kingdom; Department of Pathology, Great Western Hospitals NHS Foundation Trust, Swindon SN3 6BB, United Kingdom

**Keywords:** ovarian hyperthecosis, hirsutism, post-menopausal, hyperandrogenism, testosterone

## Abstract

We report a case of a 55-year-old postmenopausal woman who presented with symptoms of fatigue, male pattern hair loss, and hirsutism over 3 years. Investigations showed elevated total testosterone levels of 5.0 nmol/L (1.44 ng/mL; range, 0.3-3.1 nmol/L) using Beckman-Unicel-DXI-800 immunoassay. Testosterone levels were repeated by liquid chromatography-tandem mass spectrometry and were found to be elevated at 7.3 nmol/L (2.10 ng/mL). Estradiol was detectable and free androgen index was elevated. Dehydroepiandrosterone sulfate levels and androstenedione were within normal range, suggesting a nonadrenal source. Computed tomography scan of the abdomen showed no evidence of adrenal or adnexal tumor. GnRH analog stimulation test led to reduction of gonadotrophins and normalization of testosterone within 4 weeks. She had a biopsy of a cranial hair follicle, which showed androgenic alopecia. These investigations confirmed an ovarian source of androgens. Subsequently, she underwent bilateral salpingo-oophorectomy. Histological study of gonadal tissue confirmed the diagnosis of ovarian hyperthecosis. Four weeks after oophorectomy, her testosterone levels normalized and clinical symptoms improved. Ovarian hyperthecosis is a rare cause of hyperandrogenism in postmenopausal women and can pose a diagnostic and therapeutic challenge. Careful history and physical examination along with critical analysis of biochemistry and imaging studies is crucial for correct diagnosis.

## Introduction

Ovarian hyperthecosis (OH) is a condition characterized by an excess of androgen hormone production by the ovaries, resulting in symptoms of virilization such as hirsutism, acne, deepening of the voice, and increased muscle mass.

Hyperthecosis refers to the presence of nests of luteinized theca cells in the ovarian stroma because of differentiation of the ovarian interstitial cells into steroidogenically active luteinized stromal cells. This results in excessive production of androgens. The exact etiology of OH is not fully understood, but it is believed to be related to elevated levels of gonadotropins, such as LH, in postmenopausal women. The elevated LH levels can stimulate the ovarian stromal cells to differentiate into theca cells, which then produce excess androgens (1). Hyperandrogenism secondary to OH may also lead to metabolic abnormalities such as obesity and insulin resistance (2). This is also associated with a risk of endometrial hyperplasia and cancer resulting from aromatization of large amounts of testosterone to estrogen (3). Therefore, it is important to diagnose and treat OH early to prevent complications such as metabolic abnormalities and cardiovascular disease.

We report a case of a benign but relatively rare cause of hyperandrogenism in a postmenopausal woman who had a confirmed diagnosis of OH on histological examination of gonadal tissue.

## Case Presentation

We present the case of a 55-year-old woman who was referred to the endocrine clinic for symptoms of fatigue, male pattern hair loss, and hirsutism. She achieved early menopause at the age of 39 years and had a family history of early menopause. Symptoms of fatigue, hair loss, and hirsutism developed 3 years ago but significantly worsened 6 months before presentation. She had no signs or symptoms suggestive of Cushing syndrome.

Clinical parameters showed that she was obese with a body mass index of 36.4 kg/m^2^ and her blood pressure was elevated. Physical evidence suggested male pattern hair loss and hirsutism.

## Diagnostic Assessment

Initial investigations showed an elevated testosterone level of 5.0 nmol/L (144.21 ng/dL; reference range, 0.3-3.1 nmol/L) using Beckman-Unicel-DXI-800 immunoassay. Testosterone levels when repeated using liquid chromatography-tandem mass spectrometry (LC-MS/MS) were elevated at 7.3 nmol/L (210.54 ng/dL) with elevated FSH and LH consistent with her menopausal state. Dehydroepiandrosterone sulfate level was normal at 0.9 µmol/L (33.16 µg/dL; reference range, 0.0-5.0 µmol/L) and androstenedione levels were also normal at 1.9 nmol/L (54.41 ng/dL; reference range, 0.7-3.8 nmol/L). Estradiol was detectable at 66 pmol/L (17.97 pg/mL; reference range, <40.0 pmol/L) and free androgen index was elevated at 15.53% (reference range, 0.3-3.36%). Cushing syndrome was reliably ruled out by an overnight dexamethasone suppression test, which led to suppression of 0900 hours plasma cortisol level to 24 nmol/L (0.87 mcg/dL; normal response is suppression of serum cortisol to less than 50 nmol/L). Urinary steroid profile was also normal. Alpha fetoprotein and human chorionic gonadotropin levels were normal, which ruled out germ cell tumors. Full blood count, renal and liver profiles, thyroid function tests, glycated hemoglobin A1c, and 17-hydroxy progesterone were all within normal limits (Table 1).

**Table 1.** Pre- and postoperative hormone profile

Hormone tested	Pre-Surgery	Post-Surgery	Reference Ranges
Total testosterone (LS-MS/MS)	7.3 nmol/L (210.54 ng/dL)		<1.8 nmol/L (<51.90 ng/dL)
Total testosterone (Immunoassay)	5.6 nmol/L (161.51 ng/dL)	1.2 nmol/L (34.61 ng/dL)	0.3-3.1 nmol/L (8.65-89.41 ng/dL)
FSH	41.0 IU/L (41.0 mIU/mL)	38.1 IU/L (38.1 mIU/mL)	16.7-114.6 IU/L (16.7-114 IU/L)
LH	39.3 IU/L (39.3 mIU/mL)	28.2 IU/L (28.2 mIU/mL)	10.9-58.6 IU/L (10.9-58.6 mIU/mL)
Free androgen index	15.3%		0.3-3.36 %
Androstenedione	1.9 nmol/L (54.41ng/dL)		0.7-3.8 nmol/L (20.04-108.83 ng/dL)
17 Hydroxyprogesterone	1.0 nmol/L (33.04 ng/dL)		< 6.06 nmol/L (< 200 ng/dL)
Overnight dexamethasone suppression test	24 nmol/L (0.87 mcg/dL)		<50 nmol/L (1.8 mcg/dL)
Urinary steroid profile	Normal		
Alpha-fetoprotein	2.3 IU/mL (278.3 ng/dL)		0-7 IU/mL (0-847 ng/dL)
IGF-1	12.1 nmol/L (92.55 ng/mL)		6.3-27.4 nmol/L (48.18-209.58 ng/mL)
Beta-hCG	3.0 IU/L (3.0 mIU/mL)		< 5 IU/L (<5.0 mIU/mL)
SHBG	47 nmol/L (446.5 µg/dL)		30-90 nmol/L (285-855 µg/dL)

Values in parenthesis are conventional units. Abbreviations: LH, luteinizing hormone; FSH, follicle-stimulating hormone; IGF-1, Insulin-like growth factor-1; Beta hCG, beta human chorionic gonadotropin; SHBG, sex-hormone binding globulin

In view of her age and elevated total testosterone levels and further discussion with the radiology team, she went on to have an urgent whole-body computed tomography scan to rule out an androgen-producing tumor. This did not reveal any adrenal or adnexal lesions and the ovaries were reported as atrophic. However, the scan did pick up an incidental splenic aneurysm for which she was referred to the vascular team for coiling.

**Figure 1.**
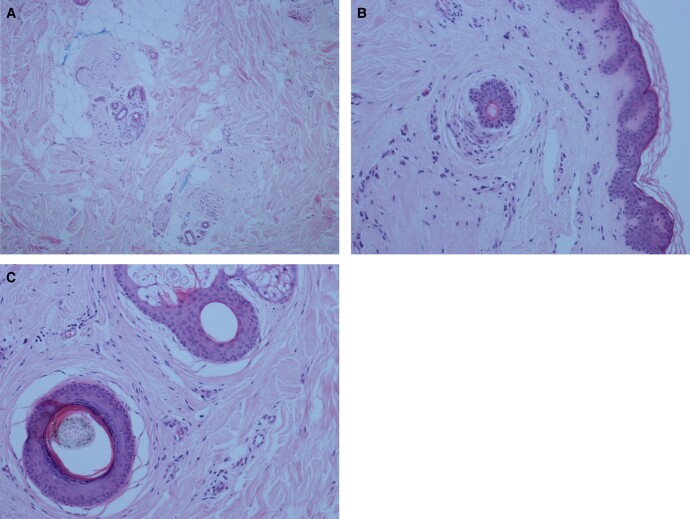
Histology of hair follicle suggesting features of androgenic alopecia. (A) ×40. There is a shift from normal terminal hairs to the much smaller vellus hairs. The site of the regressed terminal hairs is marked by fibrous follicular “stelae.” (B) ×100. The biopsies show many follicular stelae and relatively few terminal hairs. At the same time, there is an increase in vellus hairs. These changes are best appreciated by examining multiple transverse (horizontal) sections that can assess the hair follicle at various levels throughout the skin. (C) As the terminal hairs regress, the hair bulb (the deepest and most bulbous part of the hair follicle) is seen at a progressively higher level within the dermis, until it is finally replaced by a scar (follicular stelae). The diagnosis is therefore made by both qualitative and quantitative assessment. The pictures illustrate the changes recorded from the biopsy to enable diagnosis.

Interestingly, around the same time, she also had a hair follicle biopsy performed by a dermatologist privately for her frontotemporal hair loss, and the histology was consistent with androgenic alopecia (Fig. 1).

We then proceeded to perform a GnRH analogue (GnRHa) stimulation test. A single dose of GnRHa (leuprorelin, 3.75 mg subcutaneously) was administered and testosterone, LH, and FSH levels were measured before GnRHa administration, 14 days, and 28 days after GnRHa administration. Her testosterone levels suppressed significantly to 0.8 nmol/L following suppression of gonadotrophins (Table 2). This further suggested the source of androgen excess to be ovarian.

**Table 2.** Hormonal profile before GnRHa, 14 days, and 28 days after single-dose GnRHa administration

Hormone tested	Pre-GnRHa	Day 14 post GnRHa	Day 28 post GnRHa	Reference range
Testosterone	5.1 nmol/L (147.0 ng/dL)	1.8 nmol/L (51.91 ng/dL)	0.8 nmol/L (23.07 ng/dL) Level on LC-MS/MS was also 0.8 nmol/L) (23.07 ng/dL)	0.3-3.1 nmol/L (8.6-89.41 ng/dL)
FSH	41.0 IU/L (41.0 mIU/mL)	6.5 IU/L (6.5 mIU/mL)	5.4 IU/L (5.4 mIU/mL)	16.7-114.6 IU/L (16.7-114 mIU/mL)
LH	39.3 IU/L (39.3 mIU/mL)	11.6 IU/L (11.6 mIU/mL)	2.8 IU/L (2.8 mIU/mL)	10.9-58.6 IU/L (10.9-58.6 mIU/mL)
DHEAS	0.8 µmol/L (29.47 µg/dL)	0.9 µmol/L (33.16 µg/dL)	0.8 µmol/L (29.47 µg/dL)	0-5.0 µmol/L (0-184.23 µg/dL)
Androstenedione	3.3 nmol/L (94.51 ng/dL)	2.4 nmol/L (68.73 ng/dL)	1.4 nmol/L (40.09 ng/dL)	0.7-3.8 nmol/L (20.04-108.83 ng/dL)

Abbreviations: DHEAS, dehydroepiandrosterone sulfate; GnRHa, GnRH analogue; LC-MS/MS, liquid chromatography tandem mass spectrometry

## Treatment

Given that we had biochemical confirmation of an ovarian source for androgens, bilateral salpingo-oophorectomy was performed via laparoscopy, after discussion with the gynecology team. We also took into consideration the patient's perspective and preferences.

**Figure 2.**
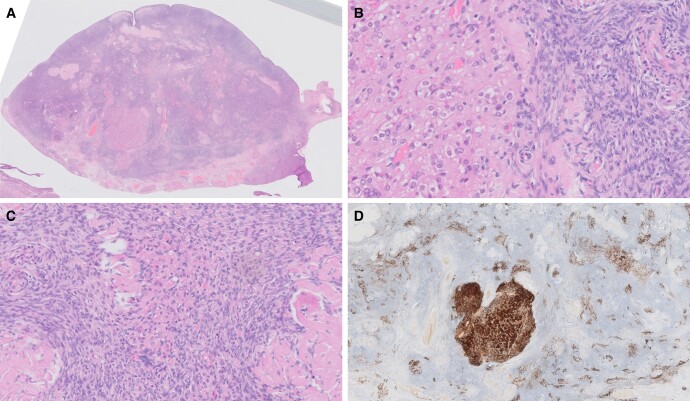
Histology of ovarian tissue displaying features of ovarian hyperthecosis. (A) Overview of the ovary showing stromal hyperplasia and loss of the normal corticomedullary interface. There is a 3-mm nodule of confluent luteinized cells; magnification × 0.5. (B) Luteinized cells with abundant eosinophilic to clear cytoplasm at the edge of the nodule; magnification × 20. (C) Small cluster of luteinized cells within the ovarian stroma (center of image); magnification × 20. (D) Immunohistochemistry for calretinin showing the luteinized cells within the nodule and in smaller clusters scattered throughout the stroma; magnification × 1.

Histological study of gonadal tissue revealed diffuse bilateral ovarian stromal hyperplasia with occasional nests of luteinized cells confirming the diagnosis of OH (Fig. 2).

Total testosterone levels reduced to normal levels (1.2 nmol/L) 4 weeks after surgery.

## Outcome and Follow-up

The patient was followed up 2 months after surgical intervention. She reported significant improvement in symptoms of fatigue and hair loss. Changes in biochemical parameters before and after treatment are summarized in Table 1.

## Discussion

This case illustrates that OH should be considered as a differential diagnosis in postmenopausal women presenting with signs and symptoms of hyperandrogenism. Careful evaluation of symptoms and investigations is essential for diagnosis. Untreated OH may be associated with increased morbidity and mortality secondary to complications of androgen excess, causing increased risk of insulin resistance which can lead to hyperinsulinemia, which in turn increases the risk of type 2 diabetes mellitus and cardiovascular disease (2, 4). Differentiating between adrenal and ovarian source of excess androgens is a crucial step in evaluating postmenopausal hyperandrogenism. Clinical course and phenotype are not reliable to differentiate between the two. Women with severe hyperandrogenemia with rapid-onset symptoms must be urgently investigated for androgen-producing tumors; therefore, it is important to promptly investigate this possibility through imaging studies. Imaging studies do not always show the origin of hyperandrogenism and occasionally may be misleading. In our case, there was no clinical or biochemical evidence of Cushing syndrome and hypothyroidism. Adrenal androgen levels were normal, which suggested a nonadrenal source of excess androgen production. Normal 17-hydroxy progesterone and urinary steroid profiles excluded adrenal disorders associated with androgen excess, including virilizing adrenal tumors and congenital adrenal hyperplasia.

We used a GnRHa stimulation test to support our diagnosis of an ovarian source of hyperandrogenism based on the principle that the secretion of androgens by ovarian tumors or OH is dependent on gonadotrophins (5‐7). Therefore, suppression of gonadotrophins by administration of GnRHa will inhibit ovarian androgen production but will have no effect on production of androgens from adrenal source. There are no current recommendations for the use of GnRHa as a diagnostic tool to help confirm an ovarian source of androgen excess. Available algorithms have recommended combined adrenal and ovarian venous sampling if imaging is inconclusive (8). This is an advanced procedure and is not offered at many facilities. Performing this procedure necessitates technical expertise, and the supportive evidence for its use in clinical practice is not yet fully established (9). It can be considered in premenopausal women contemplating pregnancy in future.

In cases where surgical intervention is not suitable and in younger women aiming for future pregnancies, GnRHa is used as a treatment modality. After administration of short-acting GnRHa, testosterone levels should fall by at least 50% from baseline value; however, after administration of long-acting GnRHa, testosterone levels are expected to fall into the reference range (5). Definitive treatment of suspected OH is by bilateral oophorectomy. Long-term GnRHa treatment can be considered as an alternative in younger women aiming to preserve fertility or if the patient is not suitable for surgical intervention (8).

## Learning Points

- Women with severe hyperandrogenemia and rapid onset of symptoms must be urgently investigated for androgen-producing tumors. This includes ovarian and adrenal imaging and careful evaluation of the hormonal profile. It is crucial to differentiate between an ovarian or adrenal source of excess androgen production.
- If testosterone levels are within the normal range using immunoassays, using liquid chromatography-tandem mass spectrometry liquid chromatography-tandem mass spectrometry should be considered if the clinical suspicion is high. Sensitivity and specificity of the liquid chromatography-tandem mass spectrometry method offer advantages over immunoassay (10).
- Administration of GnRH analog will inhibit ovarian androgen production but will have no effect on production of androgens from an adrenal source.
- Definitive treatment of ovarian hyperthecosis is bilateral oophorectomy; however, for women aiming to preserve fertility, long-term GnRH analog treatment can be offered.

## Contributors

All authors made individual contributions to authorship. R.N. was involved in the diagnosis and management of this patient. S.Y. was involved in literature review, report writing, and manuscript submission. L.J. and A.S. undertook histopathology section, legends, and preparation of histology images. All authors reviewed and approved the final draft.

## Data Availability

Data sharing not applicable to this article as no datasets were generated or analyzed during the current study.

## Abbreviations

GnRHa: GnRH analog
LC-MS/MS: liquid chromatography tandem mass spectrometry
OH: ovarian hyperthecosis
